# Predictive factors of neurologic outcome in therapeutic hypothermia after prehospital return of spontaneous circulation

**DOI:** 10.1186/cc10894

**Published:** 2012-03-20

**Authors:** Y Ohta, S Shiraishi, Y Ono, G Matsumoto, T Tagami, T Masuno, H Yokota

**Affiliations:** 1 Nippon Medical School, Tokyo, Japan

## Introduction

Induction of hypothermia is generally accepted to improve neurologic recovery of out-of-hospital cardiopulmonary arrest (CPA). Early prognostication of post-CPA patients is challenging. The aim of the present study was to evaluate the predictive factors for neurologic outcome in out-of-hospital cardiac arrest patients who returned their spontaneous circulation in a prehospital setting (PROSC) and underwent therapeutic hypothermia (TH).

## Methods

PROSC patients transported to our institution between January 2007 and May 2011 were retrospectively analyzed. TH was performed for all comatose PROSC patients admitted to the hospital for post-resuscitation care, regardless of the etiology of cardiac arrest or patient's age, except for those whose hemodynamic and pulmonary status could not be maintained. Neurological outcome at 1 month was compared as a primary end-point using the Pittsburgh cerebral performance category (CPC) scale and patients were classified into a favorable outcome group (CPC 1 and 2) or poor outcome group (CPC 3 to 5). Clinical parameters were compared between patients whose neurologic outcomes were favorable and poor.

## Results

There were 33 PROSC patients: 27 (81%) survived and 14 (42%) achieved a favorable neurological outcome. The cause of the CPA was cardiac attack in 17, noncardiac attack in 10, and unknown in six patients. Average age in the favorable recovery group was significantly younger than in the poor recovery group (62.5 vs. 70.3, *P *< 0.05). The favorable group was all the proportion of patients with ventricular fibrillation (VF) at the scene. Of the 14 that achieved a favorable neurological outcome, the cause of the CPA was cardiac attack in 12 and unknown in two patients. On the other hand, electrocardiograms of poor neurological outcome showed VF, pulseless electrical activity, and asystole. The cause of the CPA was cardiac attack in five, noncardiac attack in 10, and unknown in four. Average pH of artery blood gas (ABG) in the favorable recovery group was significantly higher than in the poor recovery group (7.31 vs. 7.17, *P *< 0.004). The receiver-operator characteristic curve for pH of ABG on arrival was analyzed. The area under the curve was 0.76.

## Conclusion

A suitable pH at the time of hospital arrival was associated with a favorable neurologic outcome among post-cardiac arrest patients without presumed noncardiac etiology.

